# Special Issue: Anti-Inflammatory Activity of Plant Polyphenols

**DOI:** 10.3390/biomedicines8030064

**Published:** 2020-03-18

**Authors:** Enrico Sangiovanni, Mario Dell’Agli

**Affiliations:** Department of Pharmacological and Biomolecular Sciences, Università degli Studi di Milano, Via Balzaretti 9, 20133 Milano, Italy; enrico.sangiovanni@unimi.it

## 1. Introduction

Inflammation is considered the first physiological response of the human body to infection or injury, playing a critical role in both innate and adaptive immunity. It is characterized by the complex biological response of vascular tissues to harmful stimuli, such as pathogens, damaged or tumoral cells, or irritants. 

Uncontrolled inflammation often results in chronic diseases, such as gastritis, arthritis, autoimmune disorders, degenerative joint diseases, rheumatisms, atherosclerosis, diabetes, and certain cancers. The inflammatory process is characterized by the migration of immune cells from blood vessels to the site of inflammation, with massive release of pro-inflammatory mediators, including cytokines, chemokines, prostaglandins, leukotrienes, and oxidative agents such as reactive oxygen species (ROS) above all.

The search for new strategies which are able to interfere with these mechanisms by preventing a prolonged inflammation would greatly benefit large number of subjects. In this respect, the plant kingdom has developed a multitude of secondary metabolites, many of which are recognized as useful tools for the maintenance of human health. Several botanicals from edible or medicinal plants are widely consumed all over the world for health purposes [1,2], including different types of 1,products, including herbal medicinal products, plant food supplements, and functional foods. 

The purpose of this Special Issue is to collect new findings on the anti-inflammatory activity of polyphenols from plants, as individual compounds or as ingredients of extracts, focusing mostly on their effects on the inhibition of pro-inflammatory mediators and their mechanism of action. 

Several polyphenols have been demonstrated to reduce the incidence of a variety of inflammatory diseases in vitro and in vivo [3] (for reviews on this topic, please refer to [4,5]).

Although the mechanism of action is strictly dependent on the class of polyphenols considered (i.e., tannins, flavonoids, lignans, etc.), several phytochemicals act as anti-inflammatory agents through the impairment of the nuclear factor κB (NF-κB) pathway, which plays a central role in the development of inflammatory response by promoting expression of adhesion molecules, cytokines and other pro-inflammatory mediators. This factor is present as an inactive form in the cytoplasm of the cells and, following stimulation by pathogens (i.e., bacteria), cytokines or pro-oxidants such as ROS, NF-κB dissociates from its inhibitory protein IκBα and translocates into the nucleus, where it modulates the transcription of a variety of cytokines and pro-inflammatory molecules. The inhibition of the NF-κB pathway, as well as its nuclear translocation, are considered key points to reduce cell injury due to chronic inflammation occurring in pathological conditions.

Several studies have confirmed that the NF-κB is among the preferential molecular target for a multitude of polyphenols (i.e., resveratrol, curcumin, genistein etc.) [6,7] or polyphenols-rich foods (i.e., olive oil, red wine etc.) [8].

Some studies have suggested that anti-inflammatory mechanisms, by which dietary polyphenols exert their positive action, involve the inhibition of C-reacting protein (CRP), as well as pro-inflammatory mediators including IL-6 and TNF α [9,10].

As an example, cocoa procyanidins have been shown to modulate the synthesis of inflammatory cytokines such as IL-1β, IL-2 and IL-6 [11] whereas procyanidins and ellagitannins from edible fruits such as chestnut (*Castanea sativa* Mill.), strawberry (*Fragaria* spp.), raspberry (*Rubus idaeus* L.) and blackberry (*Rubus fruticosus* L.) inhibit release of IL-8 by human gastric epithelial cells, and this effect is due to the inhibition of the NF-κB pathway [12,13,14]. The anti-inflammatory effect of ellagitannins for raspberry and blackberry has also been demonstrated in vivo in animal models of an ethanol-induced gastric ulcer [12].

Some of these findings have been confirmed in humans; as an example, a human clinical trial demonstrated that regular cocoa consumption decreases the serum concentrations of the pro-inflammatory endothelial adhesion molecules such as P-selectin and intercellular adhesion molecule-1 (ICAM-1), which are regulated by the NF-κB activation [15].

## 2. Recent Advances on the Role of Polyphenols in Inflammatory Conditions

This Special Issue comprises different articles reporting recent findings in the anti-inflammatory activity of plant polyphenols. The papers focused on different inflammatory conditions in a variety of cellular models, such as peripheral blood mononuclear cells (PBMC) and erythrocytes, or in vivo in animal models of glioma or ischemic optic neuropathy.

The polyphenols studied include flavonoids occurring in leafy vegetables [16], the pure molecules hydroxytyrosol (HT) and oleuropein, particularly abundant in olive oil [17], flavonoids and phenols from poplar propolis, including pinocembrin, caffeic acid phenethyl ester (CAPE) and galangin [18], anthocyanins and gallotannins from *Rhus coriaria* L. (Sumac) fruits [19].

In the last few years, the link between inflammation and nutrition has become increasingly apparent. It has been shown that excessive macronutrient intake can contribute to the inflammatory response in humans, whereas some dietary polyphenols are able to reduce the incidence of inflammatory-based diseases, including metabolic syndrome and cardiovascular disease, as confirmed by epidemiological studies [20,21]. Green leafy vegetables are important components of the human diet and are a rich source of vitamins, micronutrients, and polyphenols.

Gunathilake K.D.P.P. et al. [16] focused on the anti-inflammatory activity of six leafy vegetables widely consumed in Sri Lanka and other countries of Asia, which include plants belonging to the well-known *Cassia*, *Passiflora* and *Centella* genus. The authors demonstrated that hydroalcoholic extracts from *Cassia auriculata* or *Passiflora edulis* were able to prevent thermal and hypotonic protein denaturation by 60−70%, whereas the effect of *Centella asiatica* L. extract was found to be lower (−36%). However, most of the extracts under study showed inhibition of lipoxygenase activity by almost 50% at 100 μg/mL. According to the literature, some of the extracts considered may act through the inhibition of the release of the lysosomal constituents by neutrophyls during inflammatory processes. However, although correlation of the anti-inflammatory activity to one or more chemical entities is still premature, the occurrence of bioactive compounds, such as polyphenols, flavonoids, and carotenoids, in these leafy types may be responsible for the effects observed.

Propolis is a resinous product made by bees to repair the combs and protect the hives, preventing the microbial infection of larvae. The characteristics of propolis products are highly dependent on the vegetal origin and the geographical region in which it has been produced [22].

Governa P. et al. [18] investigated a well-characterized poplar propolis extract in PBMC stimulated with polysaccharide (LPS) from *Salmonella enteridis* to mimic bacterial infection. Micromolar concentrations of propolis significantly reduced IL-1β, IL-6, and TNFα release in LPS-stimulated human PBMC. Among individual compounds occurring in propolis, CAPE (25 μg/mL) was found to be among the major contributors to the effect exerted by propolis. The authors investigated antibacterial and antiviral activity on a H1N1 influenza A virus strain. Interestingly, propolis showed better antibacterial activity against Gram − than Gram +, with great activity against the *Streptococcus pyogenes* strain (Minimum Inhibitory Concentration, MIC: 125 μg/mL), whereas propolis did not reduce the viral load of H1N1 virus. The presence of CAPE in the formulation, although contributing to the anti-inflammatory effect, could not completely explain the biological activity observed using the phytocomplex, thus suggesting the contribution of other individual compounds, still unidentified, to the biological activity of the extract. In the literature, the effect of CAPE and propolis formulations to impair the NF-κB signalling has been previously reported, making likely the involvement of this pathway in the mode of action of propolis extracts. The results provide interesting insights into the usage of propolis, mostly in inflammatory conditions induced by pathogens of bacterial origin. 

Ramírez-Expósito et al. [17] investigated the effects of HT and oleuropein, two compounds occurring in olive oil, on the renin-angiotensin system (RAS) cascade through the proteolytic regulatory enzymes aspartyl aminopeptidase, aminopeptidase A, B, N and insulin-regulated aminopeptidase; these enzymes generate peptides which are able to regulate key processes including cardiovascular functions, body water, cell growth differentiation and proliferation, and inflammatory response. Through the use of an animal model of glioma, the authors showed a significant decrease in tumor volume in animals treated with HT following five days of treatment; moreover, treatment with HT, reduced IL-6 and TNFa production in sera of animals with glioma whereas oleuropein showed opposite effects. 

The authors concluded that the inhibitory effects on tumor growth and the anti-inflammatory status promoted by HT, but not by oleuropein or the mixture of oleuropein plus HT, may be ascribed not only to its antioxidant properties, but also to the action on different metabolic pathways/cascades, such as those mediated by the RAS or other mechanisms which regulate inflammatory processes.

Khalilpour S. et al. [19] highlighted the neuroprotective and anti-inflammatory activities of Sumac (*Rhus coriaria* L.) in a mouse model of ischemic optic neuropathy. *Rhus coriaria* is a small tree native to southern Europe, belonging to the Anacardiaceae family. The red fruits of sumac are used as a very popular spice in Persian countries, either in their pure form or in combination with other spices, due to their sour lemon taste. According to the literature, sumac fruits possess a multitude of biological activities, including beneficial effects in vivo in streptozotocin-induced diabetes [23], lipid-lowering effects in hypercholesterolemic rats [24], cardioprotective effects in hyperlipidemic patients [25], and prevention of necrotizing enterocolitis [26]. Recently, our group demonstrated the beneficial effect of *Rhus coriaria* L. fruit extracts as preventive agents in the treatment of keratinocyte inflammation through their inhibition of skin pro-inflammatory mediators, including IL-8, MMP-9, ICAM-1, and VEGF [27]. Following nerve crush injury, the mice were treated with 400 mg/kg of *Rhus coriaria* ethanolic extract, or linoleic acid (LA), which was among the main components occurring in the extract. Both the extract and pure compound decreased cathepsin activity (−84.87% and −86.71%, respectively), whose activation is strictly associated with activation of caspase-dependent cell apoptosis. The results of this study provide in vivo evidence for the neuroprotective activity of the ethanolic extract of sumac, identifying LA as the major contributor of the effect; indeed, phytochemical studies have shown that fruits are rich in hydrolysable tannins, mostly gallotannins, gallic acid derivatives, and anthocyanins, whose contribution in the biological effect of the extract cannot be excluded.

## 3. Conclusions and Future Perspectives

Recent findings on the anti-inflammatory activity of plant polyphenols clearly demonstrate that the plant kingdom is an excellent source of phytochemicals with great anti-inflammatory properties. It has been clearly demonstrated that different classes of plant polyphenols inhibit different pro-inflammatory mediators in different in vitro and in vivo models. Parameters mostly modulated by polyphenols include the impairment of the NF-κB pathway as well as the inhibition of cytokine release.

Indeed, it has to be underlined that standardization of polyphenols occurring in the extracts is mandatory to ensure the anti-inflammatory effect; moreover, the assessment of safety is another important parameter which guarantees the product quality.

Investigation aimed at identifying biomarkers in preclinical models and clinical studies which could be modulated by polyphenols are expected to generate new polyphenol-based formulations with a positive impact on quality of life in humans.
